# Fabrication of Perforated PDMS Microchannel by Successive Laser Pyrolysis

**DOI:** 10.3390/ma14237275

**Published:** 2021-11-28

**Authors:** Koungjun Min, Jaemook Lim, Ji Hwan Lim, Eunseung Hwang, Youngchan Kim, Hyunkoo Lee, Habeom Lee, Sukjoon Hong

**Affiliations:** 1 Department of Mechanical Engineering, BK21 FOUR ERICA-ACE Center, Hanyang University, 55 Hanyangdaehak-ro, Sangnok-gu, Ansan 15588, Korea; klsh6568@hanyang.ac.kr (K.M.); limjaemook@hanyang.ac.kr (J.L.); jihan9@hanyang.ac.kr (J.H.L.); joseph5017@hanyang.ac.kr (E.H.); geows3@hanyang.ac.kr (Y.K.); sean0223@hanyang.ac.kr (H.L.); 2 School of Mechanical Engineering, Pusan National University, 2 Busandaehak-ro 63 beon-gil, Geumjeong-gu, Busan 46241, Korea

**Keywords:** perforated PDMS microchannel, Glass-PDMS-Glass configuration, laser-induced pyrolysis

## Abstract

Poly(dimethylsiloxane) has attracted much attention in soft lithography and has also been preferred as a platform for a photochemical reaction, thanks to its outstanding characteristics including ease of use, nontoxicity, and high optical transmittance. However, the low stiffness of PDMS, an obvious advantage for soft lithography, is often treated as an obstacle in conducting precise handling or maintaining its structural integrity. For these reasons, a Glass-PDMS-Glass structure has emerged as a straightforward alternative. Nevertheless, several challenges are remaining in fabricating Glass-PDMS-Glass structure through the conventional PDMS patterning techniques such as photolithography and etching processes for master mold. The complicated techniques are not suitable for frequent design modifications in research-oriented fields, and fabrication of perforated PDMS is hard to achieve using mold replication. Herein, we utilize the successive laser pyrolysis technique to pattern thin-film PDMS for microfluidic applications. The direct use of thin film at the glass surface prevents the difficulties of thin-film handling. Through the precise control of photothermal pyrolysis phenomena, we provide a facile fabrication process for perforated PDMS microchannels. In the final demonstration, the laminar flow has been successfully created owing to the smooth surface profile. We envision further applications using rapid prototyping of the perforated PDMS microchannel.

## 1. Introduction

For soft lithography, in which flexible elastomers are used rather than the rigid inorganic substances, Poly(dimethylsiloxane) (PDMS) is one of the most widely used materials [1]. The inherent properties of PDMS, such as moderate stiffness, nontoxicity, optical transparency, low thermal and electrical conductivity, selective permeability, accessibility, and ease of use, allow soft lithography to have distinctive advantages over other classical lithographies in numerous fields [2,3,4,5]. Notably, the PDMS-based microchannel promises an innovative microfluidic approach that significantly impacts numerous applications [6], including biological analysis [7], microelectronics fabrication [8], analytical chemistry [9], and biological applications [10]. Moreover, the micropatterned PDMS is considered the attractive platform for a photochemical reaction regarding the enhanced reaction rate and high selectivity attributed mainly to its intrinsic light transmittance, large surface-area-to-volume ratio, enhanced mass transport, and ease of flow control [11]. In particular, these characteristics of PDMS-based microchannels could provide a promising approach for developing a process for the sphericalization of metal and ceramic nanoparticles through high-energy laser irradiation, which has recently received much attention [12,13].

However, several challenges remain to be solved for wider applications of PDMS-based microchannels. First, the low stiffness of PDMS, an obvious advantage for soft lithography, on the one hand, can be an obstacle that makes precise handling labored. In consequence, the quality of the as-prepared microchannel is greatly affected by individual operators [14,15]. For the same reason, when a certain level of pressure is applied inside or outside during operation, substantial structural deformation can occur, making it difficult to stably maintain the initial geometry and properties [16,17]. Moreover, despite the high transmittance in the spectrum from UV to NIR, the surface of PDMS can be altered by irradiated laser in that wavelength range, especially with high power density [18]. The unintended deterioration of the PDMS by the incident light can be a stumbling block to applying laser-based post-processing to materials located within the microchannel.

As a straightforward alternative free from the above problems, a Glass-PDMS-Glass (G-P-G) structure in which two glass plates with higher mechanical strength and optical stability are attached to the upper and lower side of the PDMS layer having vertically open channels can be considered. For example, due to the robust structure and low water evaporation of the G-P-G microdevice, it has been spotlighted in the PCR field [19,20,21,22]. Although research on microdevice fabrication has been continued for a few decades [23], there are several limitations in fabricating G-P-G structures through the conventional PDMS patterning techniques. First, the most widely used photolithography-based mold replica method is not suitable for frequent design modification for fast prototyping, essential at the laboratory scale because it is a complicated, time-consuming, and expensive process [24]. For the elimination of transferring PDMS film process, the main drawback of the mold-replica-based method, caramel sacrificial mold has been proposed. However, there is a backfire of additional material preparation and complex removal steps with multiple molding processes [25]. Moreover, it is difficult to prepare a PDMS layer having open channels towards both directions, i.e., perforated microchannels, through the mold replica technique requiring the transfer step of the free-standing PDMS layers to particular substrates. Recently, as a promising alternative to the mold-replica-based method, maskless laser direct patterning of a pre-cured PDMS has attracted massive interest [26,27]. For example, Shin et al. reported a successive laser pyrolysis (SLP) technique utilizing photothermal pyrolysis phenomena induced by a continuous-wave (CW) laser by which PDMS can be directly patterned even into 3D geometries [28]. The proposed method also provided smooth surfaces that are hard to be achieved by the laser ablation or burning-based direct patterning of PDMS. However, the above studies have mainly focused on modifying bulk-state PDMS rather than patterning thin-film PDMS, which is much more demanding for microfluidic applications.

Here, we examine the application of SLP using CW laser on a thin PDMS layer of 25 μm thickness formed on a glass substrate to fabricate perforated PDMS microchannels. We focused on the optimization of laser irradiation conditions for realizing the G-P-G configurations. Parametric studies on repetitive scanning and laser intensity control are performed to modify the width and depth of microchannels precisely. Finally, we demonstrated multiple channel tests using the as-prepared G-P-G microchannels successfully.

## 2. Materials and Methods

### 2.1. Preparation of G-P-G Microchannels

For preparing the lower substrate, PDMS resin is prepared by mixing the base (Sylgard 184 A, Dow Corning Corp., Midland, Michigan, USA) and a curing agent (Sylgard 184 B, Dow Corning Corp., Midland, Michigan, USA) at a weight ratio of 10:1. Then, the PDMS resin is poured onto a glass substrate (S9213, Matsunami, Osaka, Japan), followed by spin coating (ACE-200, DongAh Trade Corp., Seoul, Korea) at 2500 rpm for 60 s. The resultant sample is then cured at 70 °C for more than 1 h. For the patterning process, the PDMS layer was pyrolyzed by CW laser (Sprout-G-5 W, Lighthouse Photonics, San Jose, CA, USA) irradiating power of 0.3 W to 2.4 W focused by the objective lens (M Plan APO 2X, Mitutoyo, Kawasaki, Japan). Lower substrate and upper glass substrate with three holes were attached using oxygen plasma treatment (CUTE-3MPR/D, Femto Science Inc., Yongin, Korea). Finally, inlet and outlet tubes (AWG-11G, Banseok Precision Ind., Seoul, Korea) were inserted into the hole and sealed with silicon bond (3140 RTV, Dow Corning Corp., Midland, Michigan, USA).

### 2.2. Characterization

The top and cross-section view images in this research were obtained using an optical microscope (BX53M, Olympus Inc., Tokyo, Japan) and EBSD/FE-SEM (MIRA3, TESCAN, Brno, Czech Republic) was used to obtain microchannel top view image and contents data of carbon and oxygen.

### 2.3. Simulations

#### 2.3.1. Thermal-Induced Temperature Field Simulation

For the qualitative analysis of heat effect on the glass-PDMS interface during the SLP process, the finite element method (FEM) model was used. In detail, we derived the temperature change of three materials of PDMS, SiC, and glass during laser scanning through 2D transient simulation using COMSOL multiphysics.

#### 2.3.2. Power Absorption Simulation

We also qualitatively analyzed the power absorption on SiC surface during the multiple laser scanning. By using Lumerical finite difference time domain (FDTD) simulation, we visualized the difference of the power absorption according to incidence angle and figured out the reason for local SiC growth phenomenon with the elevation of scanning numbers at constant laser power.

## 3. Results and Discussion

### 3.1. Microchannel Fabrication Process by Front-Surface Scanning (FSS) Method

**Figure 1:**
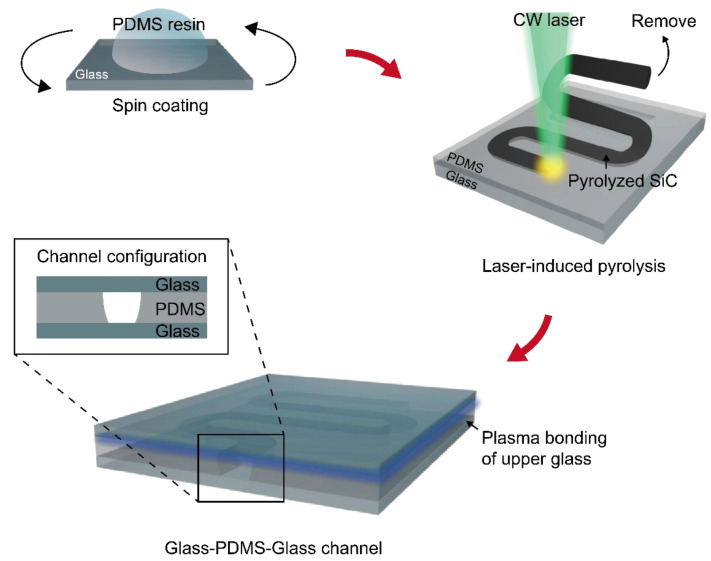
Concept illustration of the fabrication process.

Figure 1 shows the schematic illustration of the current process for the fabrication of an on-demand microchannel with a G-P-G configuration based on the SLP technique. The main objective of this process is to optimize the heat-affected zone (HAZ) created during the SLP in order to pyrolyze PDMS up to the PDMS-glass interface with minimum thermal damage at the glass surface by controlling the laser parameters precisely. While at the same time, it also should be considered that the single-layer thickness (SLT) created by a single scanning is limited due to the obstruction of the incoming laser by the SiC [28]. In this regard, a sufficiently thin PDMS layer at uniform thickness is first prepared on the glass through the spin-coating method. After the curing step, the microchannel is immediately patterned at the PDMS thin film by the FSS method that relies on the consecutive photothermal pyrolysis guided by a continuous-wave laser incident from the upper side [28]. The pyrolyzed SiC, which is a byproduct in our process, can be removed easily through the taping technique (Scotch Magic Tape, 3M, St. Paul, MN, USA) or ultrasonication. At the optimum laser conditions, the glass surface is exposed after the SiC removal without any apparent thermal damage. In order to utilize the resultant as a closed microchannel, the PDMS surface is covered by another plasma-treated glass substrate. For its application as a microfluidic device, the holes are drilled on the upper glass according to the design of the final microfluidics device before the bonding process.

The cross-sectional configuration of the resultant is presented as the inset, showing that the microchannel is created at the laser-processed region which is solely surrounded by the glass in the vertical direction. We would like to emphasize that such configuration is difficult to achieve through the conventional mold replication method [29] unless other post-etching techniques such as costly reactive ion etching (RIE) process are combined. It is also noticeable that our scheme is free from handling an ultrathin PDMS layer, i.e., detachment and relocation of the PDMS layer from the mold to the target glass substrate, which is likely to increase the chances for failure and inconsistency of the resultant device. While at the same time, the advantages of the SLP process, including smooth surface morphology and circular cross-section [28], are largely preserved in the current process.

#### 3.1.1. Laser Parameter Optimization

**Figure 2:**
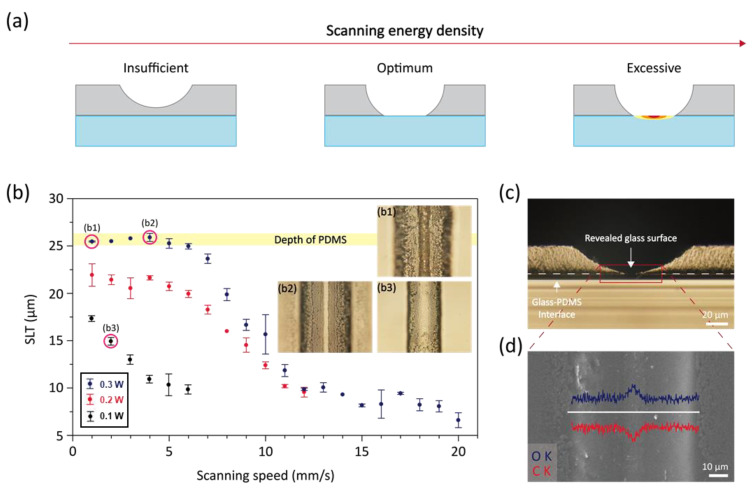
(**a**) Schematics representing substrate conditions according to scanning energy density, (**b**) Parametric study for SLT according to laser parameter and optical microscopy images of (**b1**) for excessive, (**b2**) for optimum, and (**b3**) for insufficient, (**c**) Cross-section optical microscopy image in case of (**b2**), (**d**) Top view FE-SEM image and contents data of oxygen and carbon.

Three possible outcomes from single FSS process applied to the thin PDMS layer on the underlying glass substrate are presented in Figure 2a. The single-layer thickness (SLT) of SiC created by single FSS scanning from the initiating point on PDMS is approximately proportional to the scanning energy density [28]. As a consequence, the scanning energy density has to exceed certain threshold value in order to expose the underlying glass substrate. However, at higher scanning energy density, the glass substrate can be thermally damaged due to the excessive temperature beyond its glass transition temperature. Our conjecture is that there exists optimum scanning energy density which enables complete removal of PDMS layer via SLP without bringing significant thermal impairment on the glass substrate.

Figure 2b shows the graph of the SLT created by single FSS scanning at different laser power from 0.1 to 0.3 W. The thickness of PDMS film made by spin coating has been fixed at ~25 µm, and the laser scanning speed has been controlled from 1 to 20 mm/s, which is the range that shows continuous pyrolysis of PDMS within the designated laser power. It is observable that the measured SLT is inversely proportional to the scanning speed when the SLT is <20 µm, and this result is consistent with the previous study [28]. On the other hand, the trend changes once the SLT approaches the thickness of PDMS as the thermal properties of the underlying glass substrate start to affect the temperature profile and hence the pyrolysis characteristics. As the pyrolysis progresses over 20 μm depth from the surface of PDMS to the glass substrate (25 μm distance), ~7 times higher heat conductivity of soda-lime glass (1.06 W m^−1^ K^−1^) compared to the PDMS (0.16 W m^−1^ K^−1^) alters the pyrolyzed thickness. At 0.3 W laser power, the SLP reaches the thickness of the PDMS layer when the laser scanning speed is at <5 mm/s. Because the complete pyrolysis of PDMS in the vertical direction is the prerequisite of the proposed scheme, we conclude that the threshold laser power is at ~0.3 W for the current experimental conditions. The insets in Figure 2b show the representative top-view images for each case: (b1) excessive, (b2) optimum, and (b3) insufficient scanning energy density, of which the detailed laser conditions are marked in the graph. At the optimum laser condition, the top-view shows a distinct region at the center together with sharp boundaries due to the exposed glass surface. In other cases, such region is either not clearly distinguished (b3) or covered with rough surface (b1) presumably created due to the excessive temperature at the PDMS-glass interface.

The cross-sectional optical image of the optimum condition in Figure 2c confirms that the laser-induced pyrolysis has occurred up to the PDMS-glass substrate. Because the pyrolysis front propagates into the PDMS through thermal diffusion from the absorption of the focused laser at the uppermost surface, the pyrolysis profile naturally develops into a hemispherical shape. As a consequence, the widths at the uppermost surface and at the PDMS-glass interface are not equivalent. At the optimum laser condition, the widths are measured to be 115–120 µm and 7–8 µm respectively. For further expansion of the glass-exposed region, a multiple scanning scheme has to be incorporated as explained in the following section. The clear region found in the inset (b2) of Figure 2b substantiates that the corresponding area is the glass substrate, and it is further confirmed by energy dispersive x-ray spectrometry (EDS) measurement. Carbon and oxygen contents are measured along the line perpendicular to the scanning direction and plotted in conjunction with the scanning electron microscope (SEM) image as in Figure 2d. From the compound composition of PDMS and soda-lime glass, the decrease in carbon content accompanied by the increase in oxygen content at the central region supports that the glass is exposed after the laser-induced pyrolysis and subsequent removal of the byproduct at the optimum condition.

#### 3.1.2. Microchannel Expansion through Repetitive Scanning

**Figure 3:**
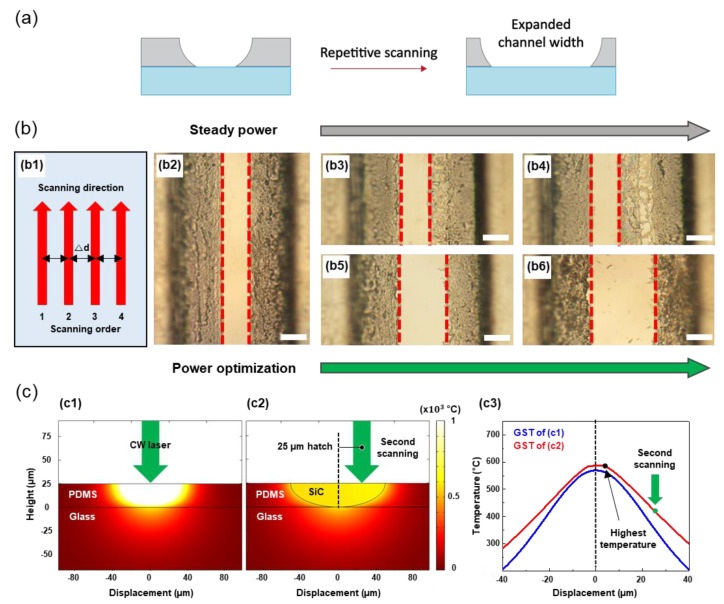
(**a**) Schematics representing cross−section image of the expanded channel, (**b**) Channel expanding order and direction shown as (**b1**), and optical microscopy images of (**b2**) for two times, (**b3**) and (**b5**) for three times, (**b4**) and (**b6**) for four times consecutively scanned samples. scale bar: 20 µm. (**c**) COMSOL simulations for thermal conduction of PDMS layer and SiC given by laser power, (**c1**) shows the result of laser irradiation on the PDMS surface, (**c2**) shows result of laser irradiation on the SiC surface with 25 µm hatched point, and (**c3**) graph shows glass surface temperatures (GST) for both cases of (**c1**) and (**c2**).

The circular cross-section enabled by SLP is often desirable for microfluidic applications [30,31], but the current widths are highly anisotropic at the uppermost layer and at the PDMS-glass interface as shown previously. The optically clear region at the PDMS-glass interface, in particular, is limited due to the pyrolysis profile created by the thermal diffusion. Because the flow characteristics in a microchannel are closely related to the channel dimensions [32,33,34], it is important to secure a method to alter the dimensions of the microchannel, and we found that carefully controlled hatching scheme [35] allows repetitive expansion of the microchannel under concern. (Figure 3a).

Using the optimal laser power and scanning speed found from the single scanning, the proper hatch distance (∆d) is firstly determined through two consecutive scans in the same scanning direction as shown in (b1) of Figure 3b. The effect of the hatch distance is investigated by analyzing the amount of nonpyrolyzed PDMS residue at the glass substrate after scanning at different hatch distances. At the optimum hatch distance of 15 µm, the optically transparent region has been increased twice as shown in (b2) compared to the result obtained from a single scanning. However, further expansion of the microchannel was unsuccessful at the identical scanning condition as it is observable from the optical images after (b3) three and (b4) four consecutive scans, showing considerable PDMS residue at the interface. Given that the scanning speed is fixed, the third and fourth scanning, therefore, require the increment in the laser power as shown in (b5) and (b6), which are the resultants from the scanning at the laser power at 0.34 W and 0.35 W for the third and fourth scanning, respectively. As a result, we expand the bottom width of the channel over seven times from 8 μm to 62 μm.

We estimate that the alteration in the optimal scanning condition at different scanning numbers is largely due to the previously converted SiC as supplemented with the series of temperature simulations (See Supplementary Note 1 for the detailed simulation conditions). Assuming that the laser initially irradiated to the PDMS layer is completely absorbed at the surface, the resultant temperature profile is predicted to be radially decreasing from the maximum temperature found at the incident spot as shown in (c1) of Figure 3c. The PDMS region subject to the temperature exceeding the threshold value is then converted into SiC, which is estimated to have a higher diffusivity compared to the pristine PDMS despite its porosity [36,37]. Due to the introduction of the SiC, the characteristics of the laser-induced temperature subject to the repetitive scanning changes significantly as shown in (c2), which is the temperature profile created by the laser irradiation 25 µm away from the center while the SiC exists. It is observable that the temperature rise happens over the SiC region instead of being concentrated at the incident spot. The temperature profiles at each case, drawn simultaneously in (c3), substantiate that the maximum temperature is not shifted as much as the hatching distance, and the laser-induced temperature at the PDMS-glass interface corresponding to the incident spot is largely limited. In summary, the expansion of the microchannel through the hatching scheme requires a precise increment in the laser power due to the enhanced thermal diffusion from the preceding SiC.

### 3.2. Microchannel Fabrication by Back-Surface Scanning (BSS) Method

**Figure 4:**
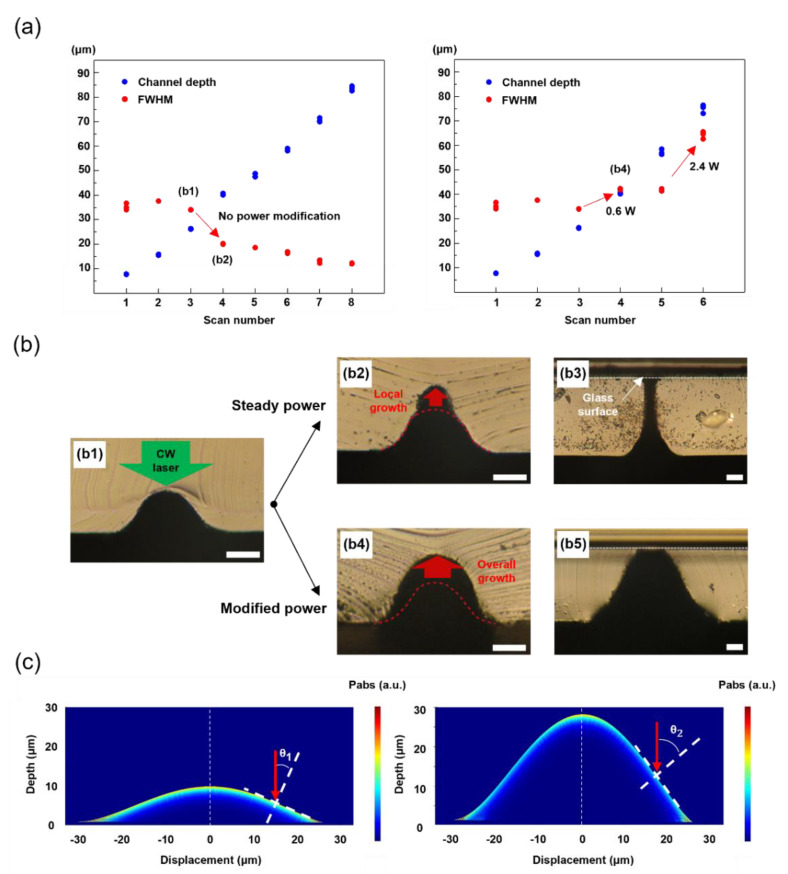
(**a**) Parametric studies of channel dimension created by BSS method according to scan numbers. (**b**) cross−section optical microscopy images of the microchannel, (**b1**) PDMS channel by 3 times of BSS, (**b2**) 4 times, (**b3**) 9 times, (**b4**) 4 times with power optimization, (**b5**) 7 times with power optimization. Scale bars from (**b1**) to (**b5**): 20 µm. (**c**) FDTD simulation result of power absorption (P_abs_).

The FSS-based method enables a direct and facile fabrication of microchannel fabrication, but it is not applicable for the fabrication of microchannel at a high aspect ratio because the SLT shows an asymptotic trend as the laser power increases [28]. The BSS method [28], on the other hand, provides a potential solution to this limitation. The main difference between BSS from FSS is that the laser beam propagates from the back through the PDMS layer. Because the outermost absorbing layer is always in contact with the PDMS in BSS, laser-induced pyrolysis occurs continuously over multiple scanning. Although it is confirmed from the previous study that the pyrolyzed depth can be increased linearly according to the scanning number [28], its shape is further characterized in this study by measuring the full-width half-maximum (FWHM) of the resultant microchannel. The pyrolyzed depth and the FWHM of the microchannel are plotted in Figure 4a. The pyrolyzed depth is in accord with the previous result [28], but it is shown that the FWHM reduces significantly as the scanning number develops. Two representative microchannels are presented in (b1) and (b2), which correspond to the optical images of the cross-sections after three and four BSS along the identical path. Compared to (b4), the pyrolyzed region grows only at the topmost region to create a more pointed microchannel, and we expect that this tendency over the scanning number is due to the incident angle-dependent reflection at the PDMS-SiC interface. Due to the hemispheric shape of the SiC, the incident angle is smaller at the topmost area, enabling low optical reflection at the interface. The FDTD simulation in (c2) further validates that the absorption and hence the heating are more intensified at the topmost region (See Supplementary Note 2 for the detailed simulation conditions). Similar to the microchannel expansion, the modification in laser power can yield different outcome in terms of FWHM. As a simple demonstration, the laser power is increased from 0.3 W to 0.6 W in (b4) at fourth scanning to suppress the sudden reduction in FWHM. It is also confirmed that the FWHM can be elevated as well by changing the laser power to 2.4 W at sixth scanning. The resultant microchannel created by (b2) and (b3) protocols, although the PDMS layers at ~90 µm are pyrolyzed completely in vertical direction in both cases, display significantly different cross-sectional profiles as shown in (b4) and (b5). Consequently, the FWHM of perforated PDMS channel is enhanced over six times from 12 μm to 79 μm as shown in Figure 4b.

### 3.3. Fabrication of Microdevice

**Figure 5:**
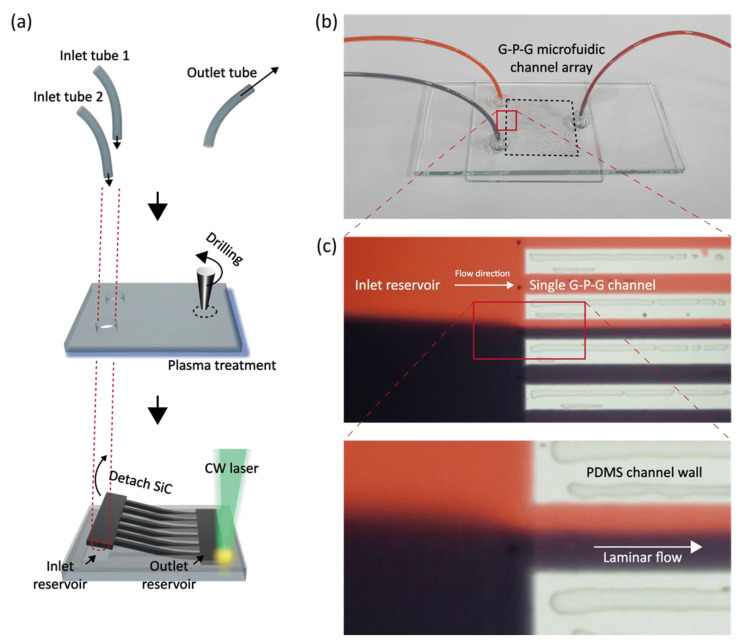
(**a**) concept illustration of microdevice fabrication, (**b**) microdevice images with tubes of inlet and outlet, (**c**) 20X magnified optical microscopy images of multiple channels showing laminar flow developed.

Because the current scheme is based on the SLP process, an arbitrary microfluidic device with a G-P-G configuration can be readily prepared at a greatly reduced tact time. A simple microfluidic device composed of two reservoirs and a series of microchannels is demonstrated as schematically shown in Figure 5a. FSS scheme has been incorporated to create the microchannels at the PDMS layer coated on the lower glass substrate, while three microholes for inlets and outlets are created separately on the upper glass substrate by using a diamond burr drill bit (Diamond Wheel Point 7134, Dremel, Racine, WI, USA). It is worth mentioning that the complete device is created within one hour, brightening the prospect for its application to rapid prototyping of customized microfluidic devices. The photograph of the final device under operation with red and black dyed water is shown in Figure 5b. Its optical microscopic images in Figure 5c validate that a laminar flow can be successfully developed in the resultant microfluidic device.

## 4. Conclusions

We introduced a facile fabrication method of G-P-G configuration microfluidic devices which is suitable for rapid prototyping on a laboratory scale. Based on the SLP technique, we pyrolyzed PDMS layer on-demand geometry of the microchannel with proper laser parameters and revealed a glass surface under the pyrolyzed area. As a result, we could eliminate PDMS film handling step, which is a labored and low-yield process, because the bottom glass substrate was not damaged during the pyrolyzing process. As a consequence of this research, we easily made a perforated PDMS microchannel which is difficult to fabricate with the mold replica method, especially in the case of thin PDMS layer. Based on the high mechanical strength and optical stability of the G-P-G configuration, we expect that the application we made be utilized in digital PCR applications and optical microfluidic systems such as biochips equipped with a high-speed camera [38,39] or laser-assisted reactive chamber usages.

## Data Availability

Data sharing is not applicable.

## Supplementary Materials

The following are available online at https://www.mdpi.com/article/10.3390/ma14237275/s1, Figure S1: Built-in settings of FDTD simulation; (**a**) ‘wavelength’ of ‘Source: Global properties’, (**b**) cross-section microscopy image of sample after 3th scanning, (**c**) geometry profile of ‘Polygon’ setting.
