# Supplementary material for: Fabrication of Perforated PDMS Microchannel by Successive Laser Pyrolysis
Source: Materials (Basel). 2021 Nov 28;14(23):7275. doi: 10.3390/ma14237275 (PMC8658685; doi:10.3390/ma14237275)
Supplement: Supplementary file 1 [file materials-14-07275-s001.zip › materials-1454775-supplementary.pdf]

materials

Supporting Information

Fabrication of Perforated PDMS Microchannel by Successive Laser Pyrolysis

Koungjun Min [1,†], Jaemook Lim [1,†], Ji Hwan Lim [1], Eunseung Hwang [1], Youngchan Kim [1], Hyunkoo Lee [1], Habeom Lee [2,*] and Sukjoon Hong [1,*]

[1] Department of Mechanical Engineering, BK21 FOUR ERICA-ACE Center, Hanyang University, 55 Hanyangdaehak-ro, Sangnok-gu, Ansan 15588, South Korea; klsh6568@hanyang.ac.kr (K.M.); limjaemook@hanyang.ac.kr (J.L.); jihan9@hanyang.ac.kr (J.H.L.); joseph5017@hanyang.ac.kr (E.H.); geows3@hanyang.ac.kr (Y.K.); sean0223@hanyang.ac.kr (H.L.)
[2] School of Mechanical Engineering, Pusan National University, 2 Busandaehak-ro 63beon-gil, Geumjeong-gu, Busan 46241, South Korea
* Correspondence: hblee@pusan.ac.kr (H.L.); sukjoonhong@hanyang.ac.kr (S.H.); Tel.: +82-51-510-2891 (H.L.); +82-31-400-5249 (S.H.)
† These authors contributed equally to this work.

Supplementary Note 1: Finite element method (FEM) simulation

Considerering heat conduction and Gaussian power distribution in transient form, 'Heat Transfer Module: Deposited Beam Power' setting was applied, expressed as

$$\left(\boldsymbol{k}\frac{\partial \boldsymbol{T}}{\partial \boldsymbol{x}}\right)+\frac{\partial}{\partial \boldsymbol{y}}\left(\boldsymbol{k}\frac{\partial \boldsymbol{T}}{\partial \boldsymbol{y}}\right)+\frac{\partial}{\partial \boldsymbol{z}}\left(\boldsymbol{k}\frac{\partial \boldsymbol{T}}{\partial \boldsymbol{z}}\right)+\mathbf{e}_{gen}=\boldsymbol{\rho}\mathbf{c}_{p}\frac{\partial \boldsymbol{T}}{\partial \boldsymbol{t}} \tag{1}$$

where k, T and e_{gen} implies conduction coefficient, temperature, and rate of energy generation given by laser power. For implementing gaussian beam scanning, 'gaussian pulse' and 'gaussian beam' settings were applied with standard deviation (σ = 4) which represents the beam intensity profile. Gaussian function is expressed as

$$\boldsymbol{Y}(\boldsymbol{x})=\frac{\mathbf{1}}{\boldsymbol{n}}\boldsymbol{e}^{\frac{(\boldsymbol{x}-\boldsymbol{x}_0)}{2\boldsymbol{\sigma}^2}} \tag{2}$$

where $\boldsymbol{x_0}$, $\boldsymbol{x}$, $\boldsymbol{Y}$ are the center of beam spot, the distance from the $\boldsymbol{x_0}$ and the beam intensity, respectively, while the normalization factor n is 1 for peak value normalization.

For the boundary conditions, room temperature (293.15 K) and natural convection coefficient (h = 5 m^2 K) is applied on the four sides. The thermal properties of PDMS (ρ = 965 kg m^{-3}, c_P = 1460 J kg^{-1} K^{-1}, k = 0.16 W m^{-1} K^{-1}), SiC (ρ = 2700 kg m^{-3}, c_P = 900 J kg^{-1} K^{-1}, k = 30 W m^{-1} K^{-1}) and glass (ρ = 2500 kg m^{-3}, c_P = 870 J kg^{-1} K^{-1}, k = 1.06 W m^{-1} K^{-1}) were applied,[1-3] where ρ and k imply the density and the thermal conduction coefficient while c_P is the specific heat capacity.

For the results of this transient simulation, the highest temperature of glass surface was obtained at 6.7 ms for the case (c1) and 6 ms for the case (c2) of figure 3c.

Supplementary Note 2: Finite-difference time-domain (FDTD) simulation

For qualitative analysis of power absorption according to incidence angle, FDTD method using Lumerical software was employed. For simulating complex geometry problem, FDTD solves Maxwell's curl equation in non-magnetic materials, expressed as

$$\frac{\partial \vec{D}}{\partial t}=\nabla\times\vec{H} \tag{3}$$

$$\vec{D}(w)=\boldsymbol{\varepsilon_0}\boldsymbol{\varepsilon_\tau}(w)\vec{E}(w) \tag{4}$$

$$\frac{\partial \vec{H}}{\partial t}=-\frac{1}{\partial \mu_0}\nabla\times\vec{E} \tag{5}$$

where D, H and E implies the displacement, magnetic and electric fields, respectively, while ετ given by the complex relative dielectric constant which is expressed as square of refractive index. [4-6] In this simulation, the solution was obtained by the built-in source setting of 'global properties', wavelength starts at 0.4 um and stops at 0.7 um shown as (a) of figure S2, and geometry setting of 'Polygon', we implemented the SiC geometry that each surface profile points were extruded from cross-section microscopy images shown as (b) and (c) of figure S1. For the input values of refractive index and extinction coefficient, the properties of carbon thin film were applied, since pyrolyzed byproduct of this process is mostly carbon. [7,8]

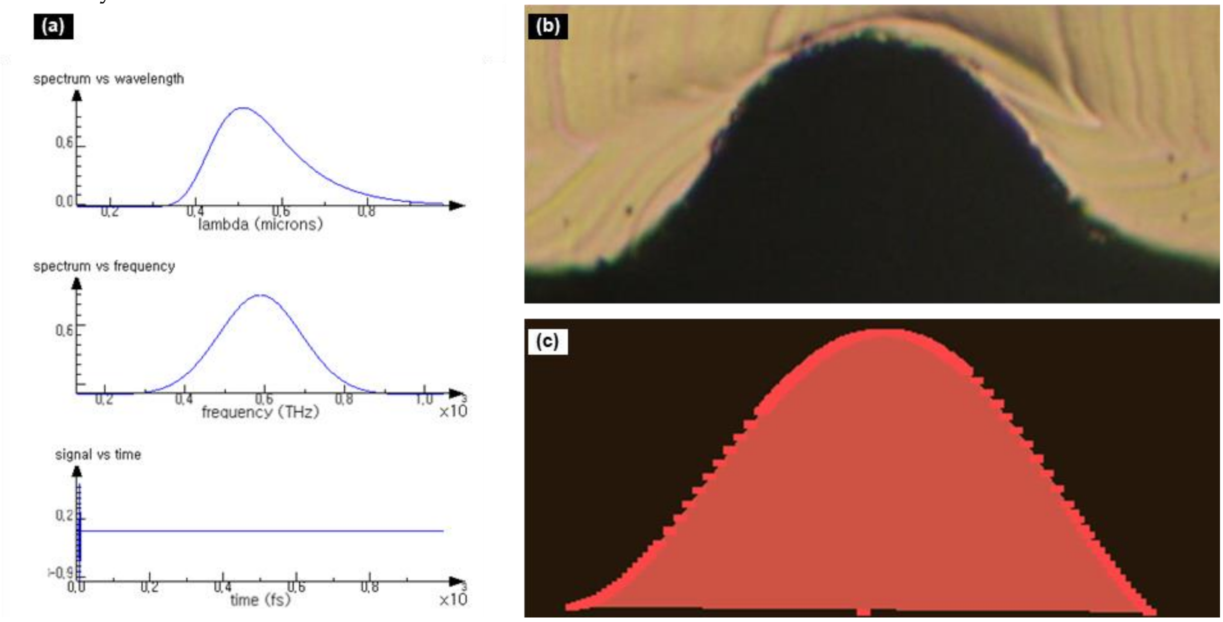

Figure S1. Built-in settings of FDTD simulation, (**a**) Wavelength, frequency settings of FDTD simulation, (**b**) cross-section image of microchannel created by back-surface scanning, (**c**) SiC geometry applied to FDTD simulation.

References

1 Santhosh, B. *et al.* Effect of pyrolysis temperature on the microstructure and thermal conductivity of polymer-derived monolithic and porous SiC ceramics. *.J. Eur. Ceram. Soc.* **2021**, *41*, 1151-1162; doi:https://doi.org/10.1016/j.jeurceramsoc.2020.09.028.

2 Wei, J. *et al.* Enhanced thermal conductivity of polydimethylsiloxane composites with carbon fiber. *Compos. Commun.* **2020**, *17*, 141-146, doi:https://doi.org/10.1016/j.coco.2019.12.004.

3 Karazi, S.M.; Ahad, I.U.; Benyounis, K.Y. in *Reference Module in Materials Science and Materials Engineering* (Elsevier, 2017).

4 Sullivan, D. M. *Electromagnetic simulation using the FDTD method*. (John Wiley & Sons, 2013).

5 Taflove, A., Hagness, S. C. & Piket-May, M. Computational electromagnetics: the finite-difference time-domain method. *The Electrical Engineering Handbook* **3** (2005).

6 Gedney, S. D. Introduction to the finite-difference time-domain (FDTD) method for electromagnetics. *Synthesis Lectures on Computational Electromagnetics* **6**, 1-250 (2011).

7 Shin, J. *et al.* Monolithic digital patterning of polydimethylsiloxane with successive laser pyrolysis. *Nature Materials* **20**, 100-107, doi:10.1038/s41563-020-0769-6 (2021).

8 Larruquert, J. I., Rodríguez-de Marcos, L. V., Méndez, J. A., Martin, P. J. & Bendavid, A. High reflectance ta-C

coatings in the extreme ultraviolet. *Opt. Express* **21**, 27537-27549, doi:10.1364/OE.21.027537 (2013).
